# The Impact of Glass Material on Growth and Biocatalytic Performance of Mixed-Species Biofilms in Capillary Reactors for Continuous Cyclohexanol Production

**DOI:** 10.3389/fbioe.2020.588729

**Published:** 2020-09-15

**Authors:** Ingeborg Heuschkel, Rakesh Dagini, Rohan Karande, Katja Bühler

**Affiliations:** Department of Solar Materials, Helmholtz-Centre for Environmental Research, Leipzig, Germany

**Keywords:** cytochrome P450 monooxygenase, pseudomonas, synechocystis, biotransformation, cyclohexane oxidation, phototrophic biofilm, continuous photobioreactor

## Abstract

In this study, the growth and catalytic performance of mixed-species biofilms consisting of photoautotrophic *Synechocystis* sp. PCC 6803 and chemoheterotrophic *Pseudomonas* sp. VLB120 was investigated. Both strains contained a cytochrome P450 monooxygenase enzyme system catalyzing the oxyfunctionalization of cyclohexane to cyclohexanol. Biofilm cultivation was performed in capillary glass reactors made of either, borosilicate glass (Duran) or quartz glass, in different flow regimes. Consequently, four phases could be distinguished for mixed-species biofilm growth and development in the glass-capillaries. The first phase represents the limited growth of mixed-species biofilm in the single-phase flow condition. The second phase includes a rapid increase in biofilm spatial coverage after the start of air-segments. The third phase starts with the sloughing of large biofilm patches from well-grown biofilms, and the final stage consists of biofilm regrowth and the expansion of the spatial coverage. The catalytic performance of the mixed-species biofilm after the detachment process was compared to a well-grown biofilm. With an increase in the biofilm surface coverage, the cyclohexanol production rate improved from 1.75 to 6.4 g m^–2^ d^–1^, resulting in comparable production rates to the well-grown biofilms. In summary, high productivities can be reached for biofilms cultivated in glass capillaries, but stable product formation was disturbed by sloughing events.

## Introduction

The ability of the microbial photosynthetic machinery to convert solar radiation into chemical energy for fixing carbon dioxide into value-added chemicals has attracted academic and industrial attention for several decades (Böhmer et al., 2017; Mellor et al., 2017). Such photo-bioprocesses are currently confined to the production of niche market products, including astaxanthin and β-carotene, with product prices within the range of 100–1,000 €/kg (Pulz and Gross, 2004; Spolaore et al., 2006; Posten, 2009; Fernandes et al., 2015; Fernández et al., 2019). The rapid progress in metabolic engineering and synthetic biology tools have broadened the available product spectrum to exploit cyanobacteria as cell factories (Angermayr et al., 2015; Hays and Ducat, 2015; Erb and Zarzycki, 2016; Gao et al., 2016; Noreña-Caro and Benton, 2018; Sun et al., 2018; Santos-Merino et al., 2019; Mukherjee et al., 2020). However, product titers and volumetric productivities obtained in these proof of concept studies have been very low compared to processes based on heterotrophic hosts such as *E. coli* or *Pseudomonas* (Liang et al., 2018; Santos-Merino et al., 2019).

Some of these challenges could be circumvented by the cultivation of phototrophic organisms in a biofilm format, where cells are naturally immobilized in a self-produced extracellular polymeric matrix. Biofilms, in comparison to suspended cells, allow long retention times for slow-growing phototrophic organisms (Elenter et al., 2007), high tolerance toward toxic chemicals (Rosche et al., 2009; Halan et al., 2016), and generate high cell density (Chang et al., 2014; Hoschek et al., 2019a), resulting in a compact reactor design and continuous operation with high volumetric productivities. In this context, a mixed-species biofilm concept, constituting phototrophic *Synechocystis* sp. PCC 6803 and heterotrophic *Pseudomonas* sp. VLB120 was established in a capillary reactor made from polystyrene (Karande et al., 2018; Hoschek et al., 2019a). This phototrophic biofilm reactor concept reported high cell densities up to 51.8 g_BDW_ L^–1^. It was successfully used for converting cyclohexane to the corresponding alcohol and resulted in 98% substrate conversion and a stable product flux of 3.76 g m^–2^ d^–1^ for one month.

For the continuous production of chemicals on a technical scale by using phototrophic biofilms, crucial factors are to obtain high and stable volumetric productivities. Additionally, the scale-up of the phototrophic capillary biofilm reactor system based on the numbering-up concept or by using glass monoliths needs to be validated (Ebrahimi et al., 2005; Kreutzer et al., 2005a,b, 2006). One major issue in this respect is the material used for the capillary biofilm reactor. In the above mentioned capillary reactor, polystyrene was used as a cheap and easy to handle material. However, it is subjected to rapid yellowing and embrittlement under UV light, limiting long-term outdoor applications (Yousif and Haddad, 2013). Additionally, this material is susceptible to deformation in the presence of organic solvents (Whelan and Whelan, 1994). Commercial photobioreactors mostly consist of glass due to its high chemical stability, non-solarizing effects during UV-light exposure, resistance to temperature variations, and exceptionally long service life up to 20 years (Burgess et al., 2007). In this work, we aim to evaluate glass as a possible material for capillary reactors and study its impact on mixed-species biofilm development and catalytic performance.

For continuous operation and stable volumetric productivities, biofilm growth and detachment need to be balanced to acquire a defined amount of active biomass within the reactors (Elenter et al., 2007; Rumbaugh and Sauer, 2020). Conversely, significant biofilm detachment events, termed as sloughing, could severely affect biofilm catalytic performance (Morgenroth and Wilderer, 2000; Telgmann et al., 2004). What fraction of mixed-species biofilm detaches in capillary reactors and how these affect the overall performance of a continuous system is not well understood. The objectives of the current work were (i) to evaluate mixed-species biofilm growth in glass capillaries under single-phase flow and segmented flow conditions (ii) to assess mixed-species biofilm stability based on the detachment process and (iii) to investigate the influence of detachment on the catalytic performance. Two glass materials, quartz and borosilicate glass were selected to study mixed-species biofilm development. The biofilm consisted of a phototrophic *Synechocystis* sp. PCC 6803 and a heterotrophic *Pseudomonas* sp. VLB120, both organisms are harboring a cytochrome P450 monooxygenase for cyclohexanol production from cyclohexane. The average cyclohexanol (CHOL) production rates of 4.72 g_CHOL_ m^–2^ d^–1^ and 4.08 g_CHOL_ m^–2^ d^–1^ could be reached for biofilms grown in quartz and borosilicate capillaries. Nevertheless, the overall biocatalytic performance for mixed-species biofilms in both glass capillaries was influenced by detachment events.

## Materials and Methods

### Chemicals

All Chemicals used for medium preparation were purchased from Carl-Roth GmbH (Karlsruhe, Germany), Merck (Darmstadt, Germany) in the highest purity available. Cyclohexane (≥99.8% purity) was obtained from Merck (Darmstadt, Germany), and cyclohexanone and cyclohexanol, ≥99.5% purity, were purchased from Sigma-Aldrich (Steinheim, Germany).

### Bacterial Strains and Plasmids

Bacterial strains and plasmids used in this study are listed in Table 1.

**TABLE 1:** Strains and plasmid used in this study.

Strain	Description	References
*Synechocystis* sp. PCC 6803	Geographical origin: California, United States; Received from Pasteur Culture Collection of Cyanobacteria (PCC, Paris, France)	Stanier et al., 1971
*Pseudomonas* sp. VLB120	Wild-type Pseudomonas	Panke et al., 1998
Plasmid		
pCyp	Based on pAH032; CYP, FdR, and FR genes under the control of Ptrc1O promoter system, RBS^∗^ optimized for Synechocystis sp. PCC 6803 in front of CYP gene	Hoschek et al., 2019b

### Cultivation of Synechocystis sp. PCC6803

Cells were grown in YBG11 medium (10 and 50 mM HEPES depending on the application) without citrate and supplemented with 50 mM NaHCO_3_, composition: 1.49 g L^–1^ NaNO_3_, 0.074 g L^–1^ MgSO_4_ 7 H_2_O, 0.0305 g L^–1^ K_2_HPO_4_, 10 mL L^–1^ YBG11 trace elements (100x), 0.019 g L^–1^ Na_2_CO_3_, 10 or 50 mM HEPES (pH 7.2); YBG11 trace elements (100x): 3.6 g L^–1^ CaCl_2_ 2 H_2_O, 0.28 g L^–1^ boric acid, 0.11 g L^–1^ MnCl_2_ 4 H_2_O, 0.02 g L^–1^ ZnSO_4_ 7 H_2_O, 0.039 g L^–1^ Na_2_MoO_4_ 2 H_2_O, 0.007 g L^–1^ CuSO_4_ 5 H_2_O, 0.005 g L^–1^ Co(NO_3_)_2_ 6 H_2_O, 0.162 g L^–1^ FeCl_3_ 6 H_2_O, 0.6 g L^–1^ Na_2_EDTA 2 H_2_O, 4.2 g L^–1^ NaHCO_3_ (Shcolnick et al., 2007).

Pre-cultures were inoculated using 200 μL of a *Synechocystis* sp. PCC 6803 cryo-stock in 20 mL YBG11 medium (50 mM HEPES) and cultivated in a 100 mL baffled shake flask at 30°C, 50 μE m^–2^ s^–1^ (LED), ambient CO_2_ (0.04%), 150 rpm (2.5 cm amplitude), and 75% humidity in an orbital shaker (Multitron Pro shaker, Infors, Bottmingen, Switzerland) for 4 days. This culture was used to inoculate a 20 mL YBG11 main culture to a starting OD_750_ of 0.08 and furthermore incubated for 4 days under the same conditions.

### Cultivation of Pseudomonas sp. VLB120

Overnight cultures were inoculated directly from a 10% glycerol stock and used for inoculation of 5 mL lysogeny broth medium (Bertani, 1951) grown at 30°C and 200 rpm (2.5 cm amplitude) in a Multitron Pro shaker (Infors). 20 mL of M9^∗^ medium (Sambrook et al., 2001) pre-cultures were inoculated (1% v/v) from this overnight culture and incubated for 24 h in 100 mL baffled shake flasks. Minimal medium main cultures were subsequently inoculated to an OD_450_ of 0.2 and grown under the same conditions for 8 h in 20 mL M9^∗^ medium.

### Pre-mixing of Bacterial Strains

The optical density of each prepared main culture (*Synechocystis* sp. PCC 6803 and *Pseudomonas* sp.VLB120) was measured and the culture completely utilized for the pre-mixing, concentrated by centrifugation (5000 × *g*, 4°C, 7 min), and resuspended in 2 mL YBG11 medium (w/o citrate, 50 mM HEPES, 50 mM NaHCO_3_, Kanamycin 50 μg mL^–1^). The mixed culture was inoculated in 20 mL YBG11 medium (w/o citrate, 50 mM HEPES, 50 mM NaHCO_3_) from the resulting cell suspensions to an OD_750_ and OD_450_ value of 1, cultivated in a 100 mL baffled shake flask and incubated at 30°C, 50 μmol m^–2^ s^–1^ (LED), ambient CO_2_ (0.04%), 150 rpm (2.5 cm amplitude), and 75% humidity in a Multitron Pro shaker (Infors) for 24 h.

### Capillary Reactor System Setup

Biofilms were cultivated in the capillary reactor system (Supplementary Figure S1A), as previously described (Heuschkel et al., 2019). Quartz (wall thickness (w.th.) 1 mm, inner diameter (i.d) 3 mm) and borosilicate glass tubes (w.th. 1 mm, i.d 3.5 mm) were cut to a length of 20 cm to serve as capillary reactors. YBG11 medium (10 mM HEPES, 50 mM NaHCO_3_) was supplied via Tygon tubing (LMT-55, 2.06 mm i.d., 0.88 mm w.th., Ismatec, Wertheim, Germany) using a peristaltic pump (ISM945D, Ismatec, Wertheim, Germany). The reactor system was inoculated by syringes through injection ports (ibidi GmbH, Martinsried, Germany) established in front of the capillary. Gas exchange for medium inlet as well as outlet and air segment generation was enabled through sterile filters (0.2 μm, Whatman, Maidstone, United Kingdom). Cultivation was performed at room temperature (RT, 24°C), and fluorescent light tubes were used as a light source (50 μmol m^–2^ s^–1^). When applied for the experiment, the air was introduced into the system via Tygon tubing connected by a T-connector in the form of air segments after 7 days of cultivation.

### Inoculation of the Capillary Reactor System

The capillary reactors were inoculated with mixed-species cell suspensions (as described in section “Pre-mixing of Bacterial Strains”) by purging 2 mL of each culture through the injection port. The medium flow was started 15 h after inoculation at a rate of ca. 52 μL min^–1^ (flow velocity of 0.09–0.12 mm s^–1^). When appropriate, air segments were started 7 days after inoculation at a rate of ca. 52 μL min^–1^ (flow velocity of 0.09–0.12 mm s^–1^), resulting in an increased overall flow rate of ca. 104 μL min^–1^ in the capillaries.

### Cyclohexane Oxyfunctionalization in Capillary Biofilm Reactors

Cyclohexane was supplied via the gas feed. Therefore, the T connector combining air feed with the medium supply was connected to a cyclohexane saturation bottle. A silicone tube was used for cyclohexane diffusion into the feed stream and was submerged in 80 mL cyclohexane in a closed 100 mL Schott glass bottle (Supplementary Figure S1B). Heterologous gene expression in *Pseudomonas* sp. VLB120_pCyp (Ps*_*CYP) and *Synechocystis* sp. PCC 6803_ pCyp (Syn_CYP) of the cytochrome P450 monooxygenase was induced after 39–41 days of cultivation by the addition of 1 mM IPTG to the medium and the cyclohexane feed was connected after one day to the capillary inlet using PTFE tubes. Liquid phase samples (1.2 mL) were collected from the reactor inlet and outlet. One mL sample was directly extracted by vigorous mixing for 2 min with 500 μL of ice-cold diethyl ether (0.2 mM decane as internal standard) followed by centrifugation (17,000 × *g*, 2 min, RT). The ether phase on top of the aqueous phase was removed, dried using anhydrous Na_2_SO_4_, and applied for quantification by gas chromatography.

### Gas Chromatography (GC) for Cyclohexane, Cyclohexanol and Cyclohexanone Quantification

Ether samples (1 μL) were injected by a PTV injector, programmed with a temperature gradient of 10°C s^–1^ from 90–300°C into a GC Trace 1310 (Thermo Fisher Scientific) equipped with a TG-5MS capillary column (5% diphenyl/95% dimethyl polysiloxane, 30 m, i.d. 0.25 mm, film thickness: 0.25 μm, Thermo Fisher Scientific). A split ratio of 11 was applied, and the oven temperature profile was set to 40°C for 1 min, 40–80°C at 10°C min^–1^, 80–320°C at 100°C min^–1^, and 320°C for 10 min, N_2_ was applied as carrier gas (flow rate: 1.5 mL min^–1^). The flame ionization detector was operated at 320°C, 350 mL min^–1^ air, 30 mL min^–1^ makeup gas, and 35 mL min^–1^ hydrogen gas flow.

### O_2_ Quantification in Gas and Liquid Phases

Dissolved O_2_ in the reactor outlet was quantified by a Clark-type flow-through sensor (OX-500 Oxygen Microsensor, Unisense, Aarhus, Denmark) connected to a microsensor amplifier (Microsensor multimeter, Unisense). When air segments were used, bubble traps (sealed with a PTFE coated silicone septum, Duran, Mainz, Germany) were attached to the capillary outlet and equilibrated for 24 h. Gas-phase (100 μL) samples were obtained from the bubble traps using gas-tight syringes (Hamilton, Reno, United States) and quantified using a Trace 1310 gas chromatograph (Thermo Fisher Scientific, Waltham, United States). The gas chromatograph was equipped with a TG-BOND Msieve 5A capillary column (30 m, ID: 0.32 mm, film thickness: 30 μm, Thermo Fisher Scientific) and a thermal conductivity detector operating at 100°C with a filament temperature of 300°C and a reference gas flow rate of 4 mL min^–1^. Argon gas was applied as carrier gas at a constant flow rate of 2 mL min^–1^. The sample injection temperature was set to 50°C with a split ratio of 2, and the oven temperature was kept constant at 35°C for 3 min.

### Determination of Biofilm Dry Weight

For biomass quantifications, the capillary reactor setup was disassembled, and the biomass removed from the tubes. The collected biomass was concentrated by centrifugation (5000 × *g*, 20°C, 7 min) in pre-dried and weight Pyrex tubes, dried again for 1 week at 80°C in an oven (Model 56, Binder GmbH, Tuttlingen, Germany) and was subsequently weighted.

## Results

### High Oxygen Amount Impedes Biofilm Development in the Single-Phase Medium Flow Conditions

**FIGURE 1:**
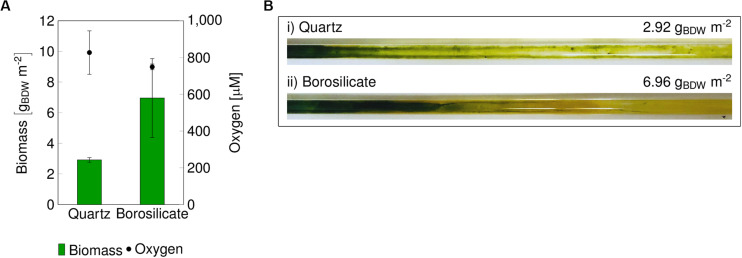
Final biomass recovered from glass capillaries, and O_2_ concentration in the reactor outlet after 14 days **(A)**. Images of the biofilm grown in quartz capillary **(B,i)** and borosilicate capillary **(B,ii)** are displayed. BDW, biomass dry weight.

The impact of the capillary material on the mixed-species biofilm development was evaluated by inoculating quartz and borosilicate glass capillaries with a mixed culture (ratio 1:1) of *Synechocystis* sp. PCC 6803 (pCyp) and *Pseudomonas* sp. VLB120 (pCyp). Two biological replicates for each capillary material were analyzed. The system was operated with a continuous feed of aqueous medium supplied at a flow velocity of 0.09–0.12 mm s^–1^ for 14 days (Supplementary Figure S2). Oxygen evolution and total biomass formation were determined at the end of the experiment (Figure 1A).

In both materials, biofilm formation followed a gradient from sufficient growth in the beginning to weak growth toward the end of the capillary. The color of the biofilm changed accordingly from dark green to yellow and reflected the viability of *Synechocystis*, which tends to “bleach” when the organism is stressed or starved. Oxygen accumulated over the capillary length and a maximum oxygen amount of 748 ± 16 μmol L^–1^ was measured in the medium phase at the end of the borosilicate capillaries. Only 6.96 g biomass dry weight (BDW) m^–2^ developed under these conditions. The saturation concentration of O_2_ in water at 25°C corresponds to 276 μmol L^–1^ indicating a pronounced oxygen oversaturation within such capillary reactors. With the highest O_2_ concentration of 827 ± 118 μmol L^–1^ in the quartz capillary, the biofilm turned to a bright yellow color with the lowest biomass content 2.92 g_BDW_ m^–2^ compared to the borosilicate capillary reactor (Figure 1B). A similar effect of high O_2_ concentrations causing a weak development of the mixed-species biofilm in polystyrene capillaries was described in the previous study (Hoschek et al., 2019a). The transition of biofilm color from light green to yellow indicates impairment of photosystem II and associated photo-pigments (Latifi et al., 2009; Hoschek et al., 2019a) due to oxygen oversaturation. Strategies to overcome oxygen stress are necessary to develop high cell density (HCD) phototrophic biofilms.

### Air Segments Relieve High Oxygen Stress but Facilitate Biofilm Flush Outs in Glass Capillaries

**FIGURE 2:**
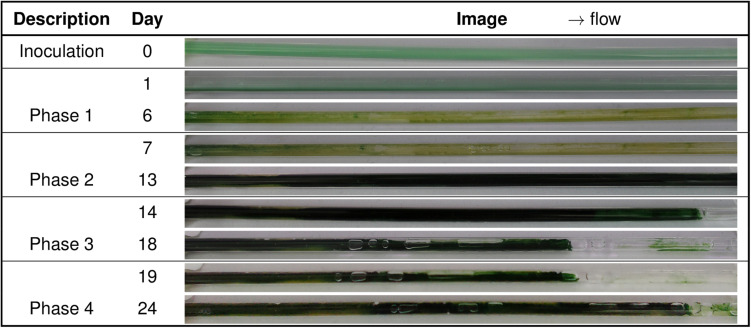
Four stages of biofilm development in a quartz glass capillary. The biofilm was grown without air segments for 7 days (phase 1) with a medium flow velocity of 0.09–0.12 mm s^– 1^, and air segments were started subsequently at the same flow rate (phase 2), which led to a fast surface coverage of the entire capillary (phase 2, 13 days total cultivation). After that, sloughing events (phase 3) and biofilm regrowth were observed (phase 4).

In both glass capillary materials, the mixed-species biofilm grown in single-phase medium flow produced high amounts of O_2_, subsequently restricting biofilm growth. In order to relieve the oxidative stress imposed by O_2_, air segments were introduced into the medium flow. Within 24 h, the biofilm started to turn from yellow to green (Supplementary Figure S3), indicating that the high O_2_ concentration was being relieved, and the impairment of the photosystem II and associated photo-pigments was reversible. The segmented flow resulted in the complete surface coverage by the dark green biofilm within 7 days (Figure 2). After the biofilm was well grown, large biofilm parts were detached in both capillaries (Figure 2). The detachment of large biofilm chunks was frequently identified at the rear part of the capillary, and the amount of detached biofilm varied profoundly (Figure 2). In these experiments sloughing events were observed after a total of 14 days of biofilm cultivation. After the detachment, the biofilm regenerated itself, which under these conditions took one to two weeks after the sloughing events to achieve maximum surface coverage.

Overall, four phases of mixed-species biofilm growth and development could be distinguished in glass capillaries. Phase 1 includes mixed-species biofilm growth under single-phase flow for 6–7 days. In this phase, the biofilm growth and development are limited due to oversaturated oxygen in the aqueous phase. In phase 2, the start of air-segments relieved oxygen stress and resulted in a rapid increase of biofilm spatial coverage. Phase 3 begins with the sloughing of large biofilm patches from well-grown biofilms. Whereas, phase 4 consists of biofilm regrowth and the expansion of the spatial coverage. When cultivated further, phases 3 and 4 were observed to be recurring.

### Stable Biocatalytic Performance of Mixed-Species Biofilms in Both Glass Materials Are Affected by Sloughing Events

**FIGURE 3:**
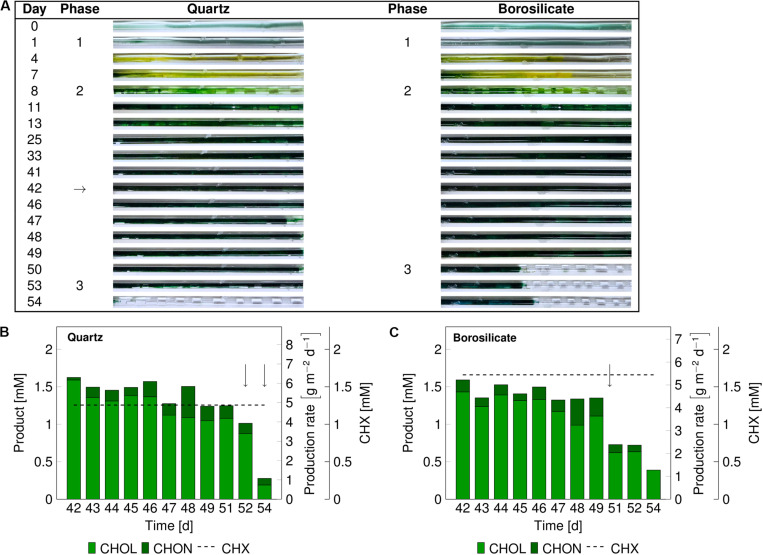
Images of the mixed-species biofilm cultivated in quartz and borosilicate glass capillaries. Flush outs were observed after 50 days of biofilm cultivation in both capillaries **(A)**. Medium and air segments (from day 8 on) were fed at a flow velocity of 0.09–0.12 mm s^– 1^. The biotransformation was introduced after 42 days (arrow panel **A**), and the product formation recorded (panels **B,C**). The cyclohexane feed is represented as a dashed line for the quartz capillary at 1.35 ± 0.42 mM and the borosilicate capillary at 1.63 ± 0.45 mM. The arrows in panels **(B,C)** indicate sloughing effects. CHX-cyclohexane.

The catalytic performance of biofilms is strongly associated with its development phases (Elenter et al., 2007). Therefore, we evaluated mixed-species catalytic performance at different biofilm developmental phases. The mixed-species biofilm was grown as described above, and the biotransformation was initiated for mixed-species biofilms at a matured state (phase 2). The product formation was measured for 13 days (Figures 3B,C). In comparison to the previous experiments (Figure 2), phase 2 was prolonged up to day 50–54 for both glass capillaries (Figure 3). The reason for this difference in the time interval is not clear.

In both capillaries, the production rate was at a maximum at the start and dropped to 1.0–1.2 g m^–2^ d^–1^ within the next 8 to 10 days. No detachments were recorded directly after starting the cyclohexane (CHX) feed. The average cyclohexanol (CHOL) production rate was 4.72 g_CHOL_ m^–2^ d^–1^ for 10 days of biotransformation in the quartz capillary and 4.08 g_CHOL_ m^–2^ d^–1^ for the first 8 days in the borosilicate capillary. The overoxidation of CHOL to cyclohexanone (CHON) was observed in both glass capillaries to be around 13%. In the quartz capillary, minor biofilm detachments were observed on day 52, leading to a drop in total product amount from 1.25 to 1.01 mM. On day 54, most parts of the biofilm were flushed out (Figure 3A), and the total production rate dropped to 0.93 g m^–2^ d^–1^.

In the borosilicate glass tube, two-third of the biofilm was detached at day 51, leading to a drop in the cyclohexanol production rate from 3.70 g_CHOL_ m^–2^ d^–1^ to 1.94 g_CHOL_ m^–2^ d^–1^. Further decrease in the production rate to 1.27 g_CHOL_ m^–2^ d^–1^ was identified on day 54. Overall, the total product formation in quartz glass was slightly higher (17%) than the borosilicate glass. Nevertheless, sloughing events were observed in both glass capillaries, resulting in a severe loss of biomass and subsequently production rates.

### Biofilm Regrowth in Borosilicate Capillaries in the Presence of Cyclohexane Leads to High Productivities

**FIGURE 4:**
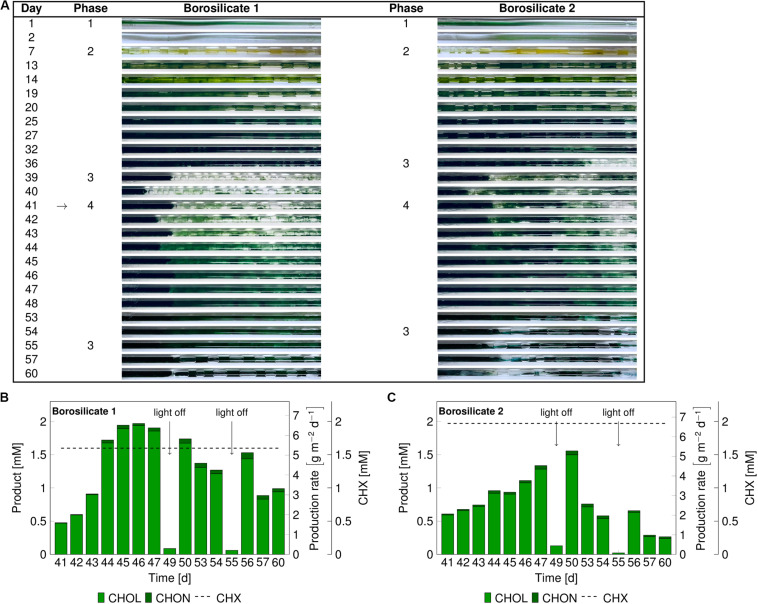
Images of the mixed-species biofilm cultivated in borosilicate capillaries **(A)**. Flush outs were found after 36 days, the biotransformation was initiated after 41 days (arrow panel **A**) and product formation recorded (panels **B,C**). Medium and air segments (from day 7 on) were fed at a flow velocity of 0.09–0.12 mm s^– 1^. The cyclohexane feed was visualized as a dashed line for capillary 1 at 1.60 ± 0.3 mM and for capillary 2 at 1.97 ± 0.70 mM. The arrows in panel **(B,C)** indicate light-off conditions. Abbreviation: CHX-cyclohexane.

In these experiments, we investigated mixed-species biofilm growth and biocatalytic performance after biofilm sloughing events (phase 3). As no major difference was observed for biofilm growth and development in both glass materials, borosilicate was chosen for further experiments. A significant portion of biofilm detached after 36–39 days (Figure 4A) due to sloughing. The biotransformation was initiated on day 41. Under the biotransformation conditions, the biofilms were able to recover completely in the following 7 days.

In the borosilicate capillary 1, the cyclohexanol production rate increased from 1.75 g_CHOL_ m^–2^ d^–1^ to 6.4 g_CHOL_ m^–2^ d^–1^, and it reached a steady-state after day 45 (Figure 4B). In this case, the system seemed to be CHX limited, as 98% of the supplied substrate was converted. In the borosilicate capillary 2, the cyclohexanol production rate increased from 1.8 g_CHOL_ m^–2^ d^–1^ to 4.89 g_CHOL_ m^–2^ d^–1^ from day 41 to 48, respectively (Figure 4C). Here, the system was not CHX limited, as only 76% of the CHX feed was converted. In both cases, minor amounts (3–4%) of the overoxidation product CHON were detected.

At day 49 and 55, the light was turned off to investigate the impact of photosynthesis on the heterologous reaction. During the first dark period, the O_2_ concentration in the air phase dropped by 5.0% and 3.7% in capillary 1 and 2, respectively (Supplementary Figure S4). Additionally, the product amount in the outflow was significantly reduced below 0.15 mM in both capillaries. Similar effects of the drop in O_2_ and CHOL production were seen during the second dark period on day 55. These results led to the conclusion that electrons provided by photosynthetic water splitting fueled the biotransformation reaction. After the regrowth of the biofilms in both capillaries under biotransformation conditions, sloughing effects were detected again. This detachment resulted in a significant drop in the product concentration on day 57 and 53 in the capillary 1 and 2, respectively. Overall, we could conclude that the catalytic performance of mixed-species biofilm is dependent on the amount of available biomass with the capillary system.

## Discussion

Cyanobacteria are considered to be promising microorganisms for converting CO_2_, water, and solar radiation into value-added chemicals (Wijffels et al., 2013; Fernandes et al., 2015; Fernández et al., 2019). The capability of cyanobacteria to assimilate CO_2_ depends primarily on the catalytic properties of the ribulose-1,5-bisphosphate carboxylase/oxygenase (Rubisco) (Tcherkez, 2016). However, Rubisco’s inability to discriminate between O_2_ and CO_2_ results in carboxylation and oxygenase activity, depending on their concentrations and the kinetic parameters (Liang et al., 2018; Flamholz et al., 2019). As oxygen is continuously generated in the oxygenic photosynthesis during the light reaction, its amount within the photosynthetic cell is considered to be higher than the ambient oxygen level (Kihara et al., 2014). Excess oxygen concentration in the functional photosynthetic cells not only affects the net carboxylation rate but can form reactive oxygen species (ROS), leading to oxidative damage of DNA, lipids, and proteins. In contrast to the suspended format, cyanobacterial cells growing in biofilms or microbial mats could reach oxygen concentrations several times higher (ca. 1000 μM) than the air-saturated water resulting in oxygen bubble formation (Jorgensen et al., 1983; Revsbech et al., 1983; Bosak et al., 2010). Correspondingly, we observed high oxygen amount of 748 to 827 μM in the reactor outflow (Figure 1). Under such high oxygen content, the development of the mixed-species cyanobacterial (Syn. sp. PCC6803) biofilms were severely affected, leading to low biomass concentration in the glass capillaries.

In the previous work, high oxidative stress was overcome by introducing air segments, utilizing citrate catabolism in *Pseudomonas* species, and an oxygen-dependent biotransformation reaction to enable HCD mixed-species phototrophic biofilms in polystyrene capillary reactor (Hoschek et al., 2019a). In this work, air segments were introduced into the medium flow to extract excess O_2_ from the biofilm and thereby to relieve oxidative stress imposed onto the cells. Overcoming the stress resulted in thick and dense biofilm growth (Figure 2). However, the mixed-species phototrophic biofilm growth and development in the glass capillaries showed four distinct phases based on the investigated time frame as compared to only two phases in polystyrene capillary reactors (Hoschek et al., 2019a).

### Biofilm Sloughing: A Critical Issue in the Development of Stable Mixed-Species Biofilm

Biofilm development is characterized by constant removal of cells from the biofilm either when small portions of the biofilm are lost by frictional forces (erosion) or when large fractions are lost based on sloughing events (Rumbaugh and Sauer, 2020). Sloughing is considered to be an integral part of biofilm development (Telgmann et al., 2004), which could lead to a loss of more than 90% biomass within one day (Figure 2). A similar event was described by Telgmann et al. (2004), where 80% of biomass was lost due to the detachment of large biofilm portions. Reasons for sloughing are widespread. These include shear stresses, nutrient to oxygen gradients as well as a change in c-di-GMP levels inside the biofilm, e.g., by c-di-GMP-degradation (Hunt et al., 2004; Thormann et al., 2006; Stewart and Franklin, 2008; Rumbaugh and Sauer, 2020). In the mixed-species biofilm, the change in biomass and thickness with the biofilm growth (phase 2) in glass capillaries could increase (aqueous-air) segmented flow velocity and subsequently intensify shear stresses and aqueous-air interfacial forces. These external fluidic forces might become more substantial than the biofilm adhesive forces leading to the sloughing or detachment (Stoodley et al., 2002; Horn et al., 2003; Sharma et al., 2005; Paul et al., 2012). However, such events were not frequently observed in polystyrene capillaries (Hoschek et al., 2019a). This outcome points out that mixed-species biofilms have weakly adhered to glass capillaries as compared to the polystyrene capillary. There is also a possibility that biofilm adhesion to the glass surfaces becomes weaker with growth due to nutrient starvation or a different stress response of the biofilm. In the mixed-species biofilm, we observed yellow locations at the bottom of biofilm (Figure 2). These color changes (green to yellow) might indicate nutrient starvation or oxidative stress because of high local O_2_ concentrations. Such stresses might weaken the cell attachment and overall biofilm structure, leading to biofilm dispersion and subsequent detachment (Ohashi and Harada, 1995; Bazin and Prosser, 2018). Overall, the increase of external fluidic forces with biofilm growth and the decrease of internal biofilm strength caused by the hydrolysis of the polymeric biofilm matrix or due to oxidative stresses could be possible reasons for biofilm sloughing.

### Biofilm Sloughing Severely Affects Mixed-Species Catalytic Performance

Sloughing may have two different effects on the product formation rate: (I) No impact on reactor performance, when only inactive biomass is lost, (II) severe impact on reactor performance due to the removal of active biomass (Elenter et al., 2007). In our experiments, sloughing was observed after the mixed-species biofilm reached a well-grown or matured stage, and this detachment was accompanied by a severe drop in the catalytic activity (Figures 3, 4). These results conclude that the lost biofilm consisted mainly of active biomass. An average production rate of 4.72 g_CHOL_ m^–2^ d^–1^ (7.18 g L^–1^ d^–1^) could be reached for a maximum of 10 days for the biofilm grown in the quartz glass capillary (Table 2).

**TABLE 2:** Comparison of literature data for cyclohexanol production from cyclohexane.

Biofilm	Capillary material	Average space-time yield [g_CHOL_ m_tube_^–2^ d^–1^] ([g_CHOL_ L_tube_^–1^ h^–1^])	Stability [d]	Electron donor	Remark	References
Pseudomonas sp. VLB 120	Silicone tube	4.8 (0.4)	18	Yeast extract	Organic carbon	Karande et al., 2016
Mixed species^a^	Polystyrene	3.8 (0.2)	31	Water	Minimal medium, no organic carbon	Hoschek et al., 2019a
Mixed species^a^	Quartz glass	4.72 (0.3)	10	Water	Minimal medium, no organic carbon	This study
Mixed species^a^	Borosilicate glass	4.08 (0.2)	8	Water	Minimal medium, no organic carbon	This study

*^*a*^Mixed species biofilm *Synechocystis* sp. PCC6803 and *Pseudomonas* sp. VLB120.*

Still, for a continuous biofilm-based process, 10 days of stable biocatalytic performance is considerably short. Our previous study reported a stable production rate of 3.8 g_CHOL_ m^–2^ d^–1^ for 31 days in a capillary reactor made from polystyrene (Karande et al., 2018; Hoschek et al., 2019a). In comparison to polystyrene capillaries, the glass surface has little texture, with an average roughness of 100 nm (for borosilicate glass) (Preedy et al., 2014). Here, a rougher surface might promote biofilm attachment and resistance against hydrodynamic forces to minimize sloughing events (Picioreanu et al., 2000). Furthermore, the light focusing effect of the glass-capillaries (Posten, 2009) might lead to increased light input resulting in high photosynthetic activity and enhanced O_2_ accumulation. Such high oxygen content could trigger sloughing events and, therefore, biofilm growth and development at different light intensities (low 25 μE m^–2^ s^–1^, high 100 μE m^–2^ s^–1^) or with light-dark cycles need to be investigated to minimize sloughing events and retain constant production rates in glass capillaries.

## Conclusion

We observed four-phases for mixed-species biofilm growth in the glass-capillaries. It comprises biofilm growth, detachment, and regrowth. The change in flow condition from single to aqueous-air segmented flow resulted in faster growth, improved surface coverage, and enhanced biomass formation. For mature biofilms, biofilm detachment via sloughing and regrowth was frequently observed in glass capillaries. The biocatalytic performance of mixed-species was evaluated at different developmental phases. For mature biofilms, an average production rate of 4.72 g_CHOL_ m^–2^ d^–1^ for 10 days and 4.08 g_CHOL_ m^–2^ d^–1^ for 8 days were obtained for quartz and borosilicate glass, respectively. Product formation was associated with biofilm biomass and increased with the re-growing biofilm. Maximum product formation of 6.5 g m^–2^ d^–1^ was observed in the borosilicate capillary, although both glass types showed comparable results. The presence of the toxic substrate cyclohexane did not hamper biofilm growth and spatial coverage. The utilization of glasses as capillary reactor materials offers several benefits, such as the light focusing effect and excellent stability against solvents or UV radiation. Nevertheless, sloughing events were observed to be higher compared to other capillary materials, e.g., polystyrene (Hoschek et al., 2019a). Therefore, future research efforts on understanding sloughing mechanisms in the mixed-species biofilms and finding solutions to minimize them in glass capillaries are necessary.

## Data Availability Statement

The raw data supporting the conclusions of this article will be made available by the authors, without undue reservation.

## Author Contributions

RD and IH planned and conducted the experimental work and analyzed data. IH wrote the manuscript. RK and KB participated in designing experiments, data analysis, and correcting the manuscript. All authors contributed to the article and approved the submitted version.

## Conflict of Interest

The authors declare that the research was conducted in the absence of any commercial or financial relationships that could be construed as a potential conflict of interest.
