# Supplementary material for: The Impact of Glass Material on Growth and Biocatalytic Performance of Mixed-Species Biofilms in Capillary Reactors for Continuous Cyclohexanol Production
Source: Front Bioeng Biotechnol. 2020 Sep 15;8:588729. doi: 10.3389/fbioe.2020.588729 (PMC7522790; doi:10.3389/fbioe.2020.588729)
Supplement: Supplementary file 1 [file Data_Sheet_1.ZIP › Supplementary_Material/SupplementaryMaterial.pdf]

Supplementary Material

1 SUPPLEMENTARY FIGURES

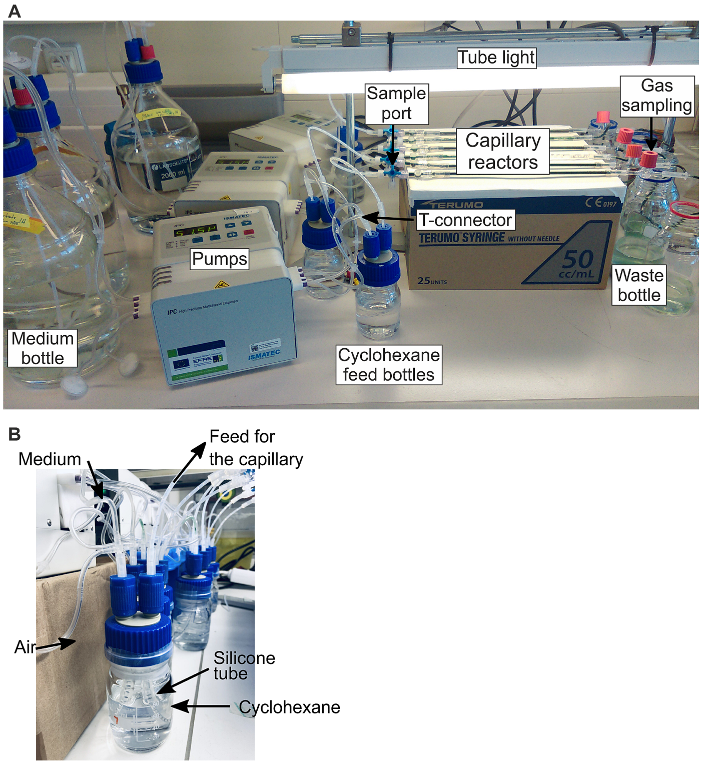

Figure S1. Shown is the cultivation setup **(A)** as well as the cyclohexane feed **(B)**. For the cyclohexane feed a silicone tube is submerged into pure cyclohexane, which allows diffusion. Medium as well as air segments were passed through this tube to get saturated with cyclohexane and fed directly into the capillary reactors.

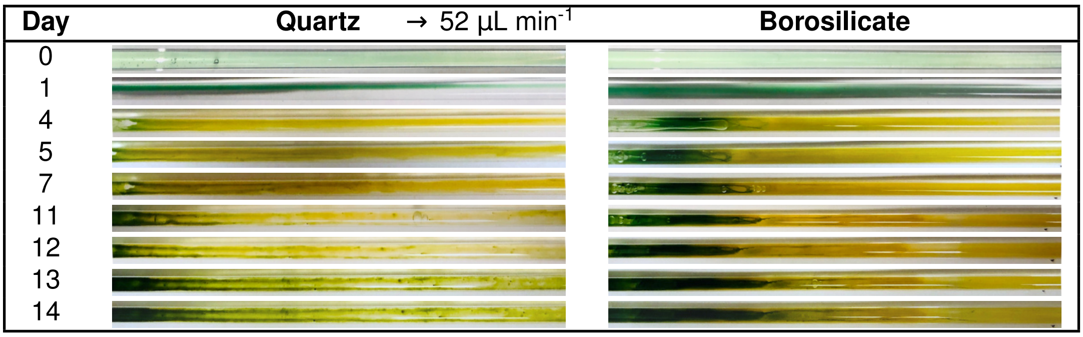

Figure S2. Images of the biofilm growth for 14 days in glass capillaries.

Description	Image
6 days medium flow	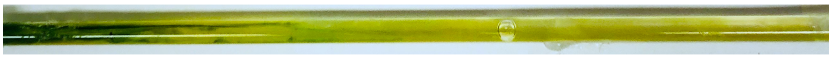
24 h with air segments	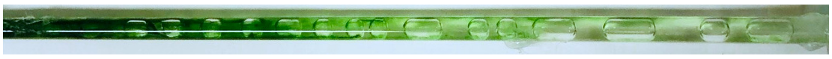

Figure S3. The mixed-species biofilm was cultivated for 6 days in a borosilicate capillary in single-phase medium flow. After the sixth day air-segments were started and the colour turned from yellow to green in 24 h.

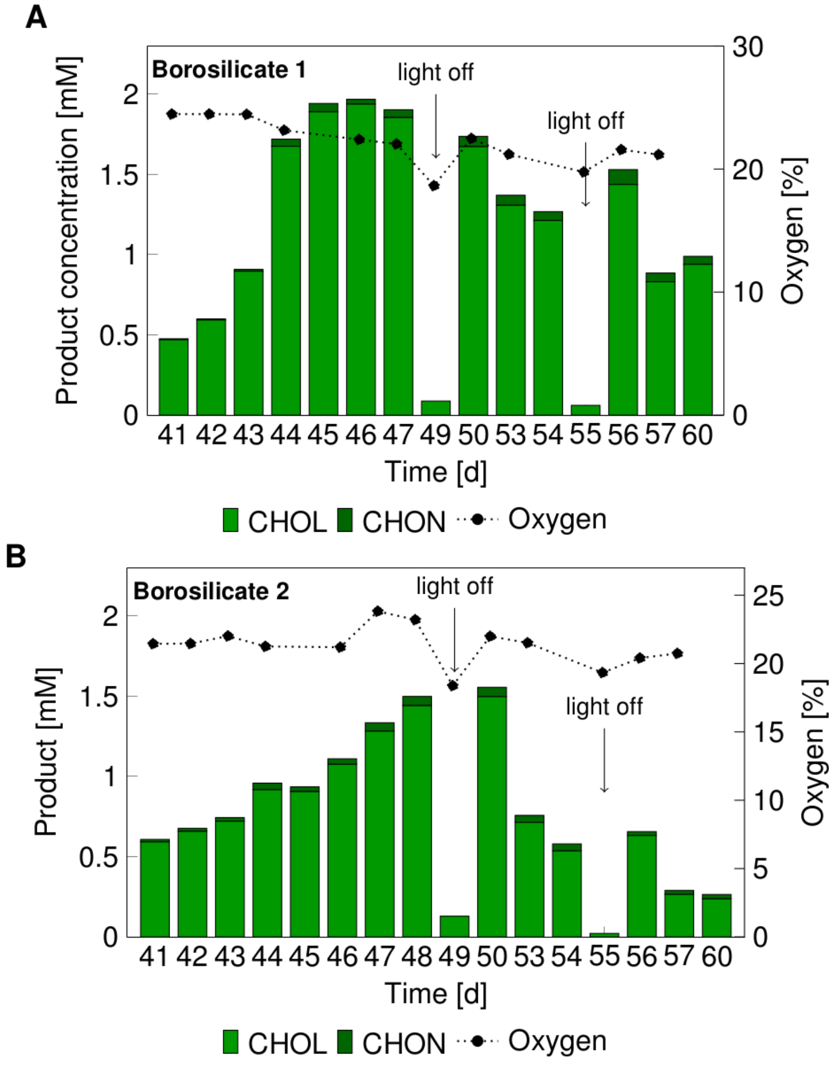

Figure S4. Oxygen outlet concentration of the biotransformation shown in Figure 4 of the main manuscript. Panels **(A)** and **(B)** visualize product formation for biofilm grown in two independent borosilicate capillaries. Arrows indicate the light off condition.
