# Supplementary figures and images for: The Impact of Glass Material on Growth and Biocatalytic Performance of Mixed-Species Biofilms in Capillary Reactors for Continuous Cyclohexanol Production
Source: Front Bioeng Biotechnol. 2020 Sep 15;8:588729. doi: 10.3389/fbioe.2020.588729 (PMC7522790; doi:10.3389/fbioe.2020.588729)

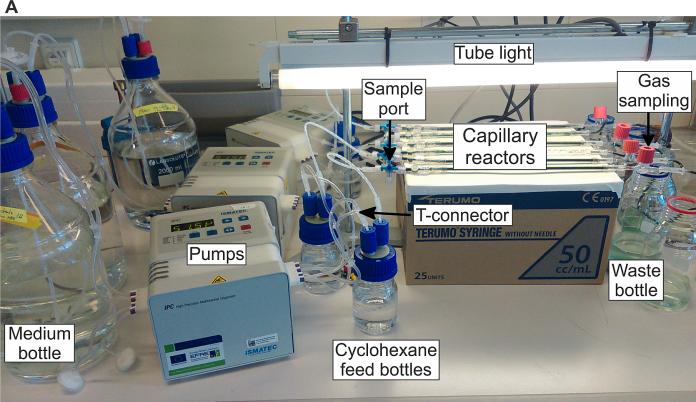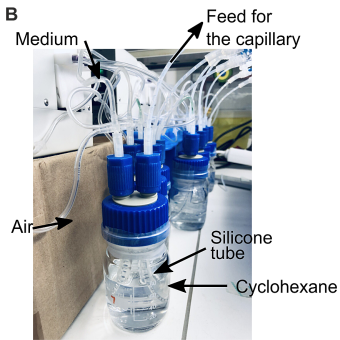

Supplement: Supplementary file 1 [file Data_Sheet_1.ZIP › Supplementary_Material/Images/Fig_S1.pdf]

| Day | Quartz                                                                              | → 52 $\mu\text{L min}^{-1}$ | Borosilicate                                                                         |
|-----|-------------------------------------------------------------------------------------|-----------------------------|--------------------------------------------------------------------------------------|
| 0   | 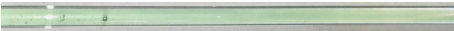  |                             | 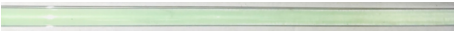  |
| 1   | 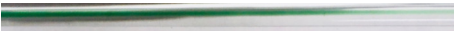  |                             | 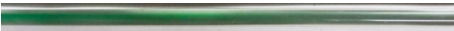  |
| 4   | 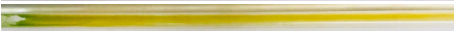  |                             | 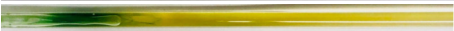  |
| 5   | 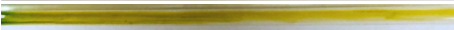  |                             | 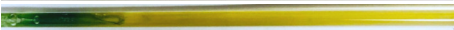  |
| 7   | 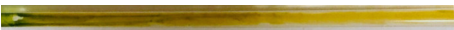  |                             | 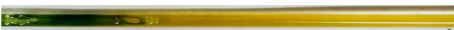  |
| 11  | 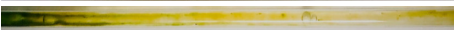  |                             | 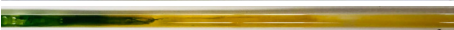  |
| 12  | 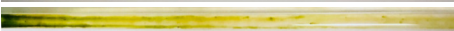  |                             | 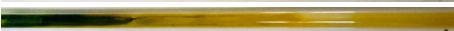  |
| 13  | 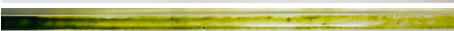  |                             | 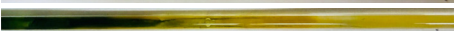  |
| 14  | 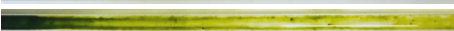 |                             | 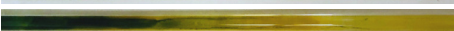 |

Supplement: Supplementary file 1 [file Data_Sheet_1.ZIP › Supplementary_Material/Images/Fig_S2-eps-converted-to.pdf]

## Description

6 days medium flow

## Image

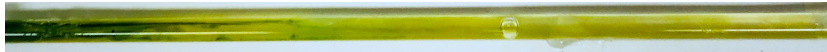

24 h with air segments

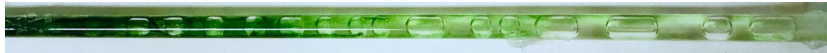

Supplement: Supplementary file 1 [file Data_Sheet_1.ZIP › Supplementary_Material/Images/Fig_S3.pdf]

**A**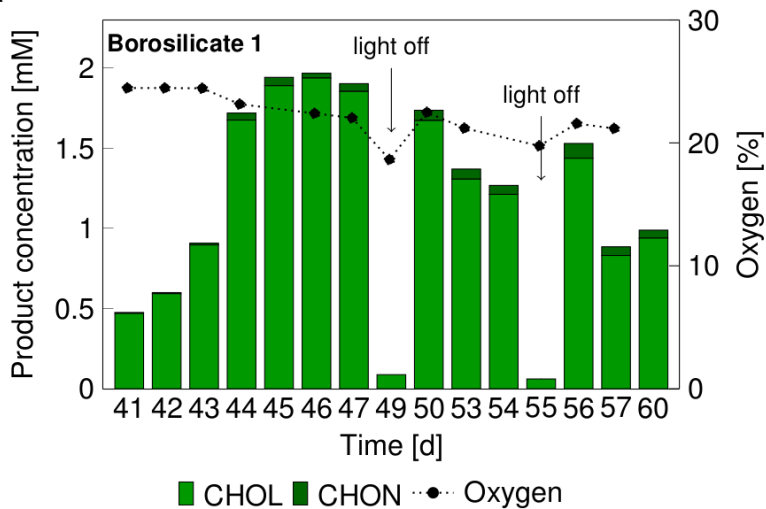**B**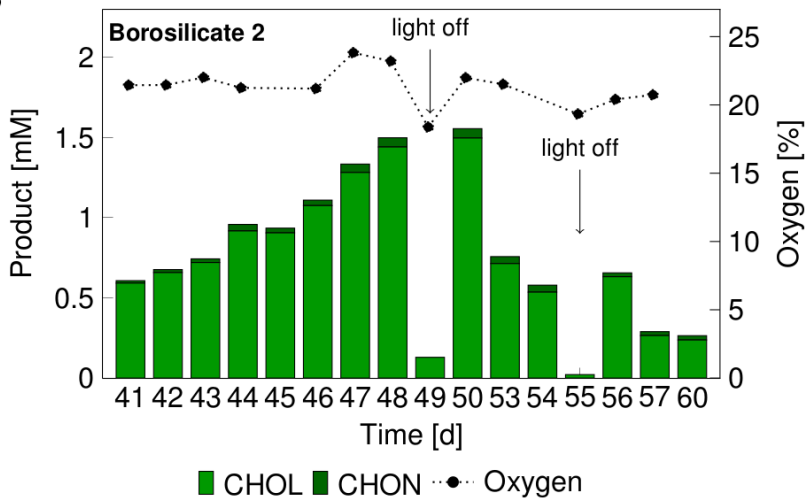

Supplement: Supplementary file 1 [file Data_Sheet_1.ZIP › Supplementary_Material/Images/Fig_S4.pdf]

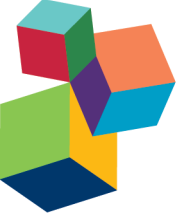

frontiers

Supplement: Supplementary file 1 [file Data_Sheet_1.ZIP › Supplementary_Material/Images/logo1-eps-converted-to.pdf]

A

frontiers  
FOR YOUNG MINDS

B

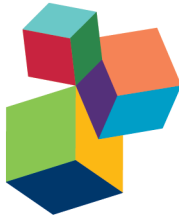

Supplement: Supplementary file 1 [file Data_Sheet_1.ZIP › Supplementary_Material/Images/logos-eps-converted-to.pdf]
